# Combining respondent-driven sampling with a community-based participatory action study of people who smoke drugs in two cities in British Columbia, Canada

**DOI:** 10.1186/s12954-021-00482-8

**Published:** 2021-03-26

**Authors:** Sulaf Elkhalifa, Ehsan Jozaghi, Samona Marsh, Erica Thomson, Delilah Gregg, Jane Buxton, Ann Jolly

**Affiliations:** 1 School of Public Health, Room 101, 600 Peter Morand Crescent, University of Ottawa, Ottawa, ON KIH 8M5 Canada; 2 School of Population and Public Health, University of British Columbia, 2206 East Mall, Vancouver, BC V6T 1Z3 Canada; 3 Vancouver Area Network of Drug Users, 380 East Hastings Street, Vancouver, BC V6A 1P4 Canada; 4 British Columbia/Yukon Association of Drug War Survivors, 380 East Hastings Street, Vancouver, BC V6A 1P4 Canada; 5 Sex Workers United Against Violence, 334 Alexander Street, Vancouver, BC V6A 1C3 Canada; 6 The Fraser Health Authority, Suite 400, Central City Tower, 13450 – 102nd Avenue, Surrey, BC V3T 0H1 Canada; 7 University of Fraser Valley, 33844 King Road, Abbotsford, BC V2S 7M8 Canada; 8 Western Aboriginal Harm Reduction Society, 380 East Hastings Street, Vancouver, BC V6A 1P4 Canada; 9 British Columbia Centre for Disease Control, 655 West 12th Avenue, Vancouver, BC V5Z 4R4 Canada; 10 Ottawa Public Health, 100 Constellation Drive, Ottawa, ON K2G 6J8 Canada; 11 UBC Faculty of Dentistry, Nobel Biocare Oral Health Centre, 2151 Westbrook Mall, Vancouver, BC V6T 1Z3 Canada

**Keywords:** Harm reduction, Respondent-driven sampling, Social networks, Naloxone, Street drugs, Substance-related disorders

## Abstract

**Background:**

The smoking of illicit drugs presents a serious social and economic burden in Canada. People who smoke drugs (PWSD) are at increased risk of contracting multiple infections through risky drug practices. Peer-led harm reduction activities, and the resulting social networks that form around them, can potentially minimize the dangers associated with the smoking illicit drugs.

**Goal:**

The goals of this study were to pilot test the combined approaches of respondent driven sampling with community based participatory action research in these settings and compare the attributes and social networks of PWSD in two British Columbia cities with different harm reduction programs.

**Methods:**

Using community-based participatory action research (CBPAR) and respondent-driven sampling (RDS), individuals with lived drug experiences were employed from communities in Abbotsford and Vancouver as peer researchers to interview ten contacts from their social networks. Contacts completed a questionnaire about their harm reduction behaviours and interactions.

**Results:**

We found that PWSD residing in Abbotsford were more likely to report engaging in harm-promoting behaviours, such as sharing, reusing, or borrowing crack pipes. However, PWSD in the Downtown East side Community of Vancouver were more likely to report engaging in harm-reducing activities, such as being trained in naloxone use and CPR. We found no differences in network sizes between the two communities, despite the population differences and harm reduction programs

**Conclusion:**

The high participation rates and interactions between researchers, and peer researchers enriched the study implementation and successfully informed our results. The fact that there were no differences in network size suggests that people have similar support in Vancouver as in Abbotsford, and that drug use practices differ mainly due to availability of harm reduction programming and resources.

## Introduction

Illicit drug use has been the focus of much concern in recent years. In Canada, 4% of people over 15 and older reported that they experienced harm resulting from illegal drug use in 2017, a 1% rise from the previous study in 2015. These estimates exclude the use of cannabis. The prevalence of harm was greater in people who reporting using both illegal and psychoactive drugs. Additionally, harm reported by women who used both, rose substantially from 11% of women in 2015 to reported harm compared to 18% in 2015 [1]. Sustained and chronic use of drugs has been linked to multiple adverse health events resulting in death and disability [2, 3].

In Canada, several harm-reduction approaches have been adopted to address the dangers surrounding the consumption of drugs. In contrast to traditional drug cessation programs, harm reduction programs aim to mitigate harmful drug-related behaviours without requiring people who use drugs (PWUID) to abstain from drugs [4]. Effective programs often employ peers (individuals with lived drug experiences) to design, implement, and deliver activities [5].

Harm reduction measures can have different forms. The two most popular are the needle exchange programs (NEP) and supervised consumption facilities (SCF) [4]. While these programs have been found to alleviate the harms associated with intravenous drug use, less attention and fewer resources have been allocated to people who smoke drugs (PWSD) [6–8]. PWSD who engage in unsafe drug practices are at increased risk of contracting bloodborne and pulmonary infections [9, 10] DeBeck et al. found that the sharing and reuse of drug paraphernalia is a significant mechanism by which pathogens are transmitted between smokers. Specifically, HIV and other bloodborne pathogens are conveyed to and from pipes via oral wounds and sores [11]. In Vancouver, Canada, a survey of PWSD found that approximately half of surveyed participants reported sharing crack pipes within the previous six months [12].

The Downtown Eastside Community (DTES) of Vancouver has a large population of drug users [13] and the largest proportion of the city’s drug arrests [14]. Consequently, it has been the focus of multiple harm reduction programs. Vancouver’s DTES community has functioned as a focal point for Canadian drug research for many years and has received a great deal of resources. The total cost of the services and programs available to this fifteen-square-block community has been estimated at more than $1 M per day. In 2013, 260 social and non-profit agencies were operating in the community, totalling $360 M per year [13]. At the forefront of these operations is the Vancouver Area Network of Drug Users (VANDU), an organization comprising individuals with lived drug experiences who elect a board to represent them. Peer-led activities offered by VANDU include supervised drug consumption facilities, and needles and crack pipes distribution [15]. One of us, (EJ) had worked with VANDU on a previous project [16] and one of the board members had suggested a study of social networks of people who smoke drugs as many obtained pipes from VANDU but not much was known about them. A proposal was developed and planning meetings with VANDU continued in advance of the project.

An organisation similar to VANDU was being established in rural Abbotsford, British Columbia. However, in contrast to the DTES, peer-led harm reduction activities in Abbotsford were not so well funded, organisation in the community was more recent, and programs were limited in scope. The initial motivation to include Abbottsford came from a VANDU board member, who suggested including it due to the lack of services in that underserved, rural area.

In this study, we wished to establish the feasibility of combining community based participatory research with respondent driven sampling, and compare the experience of peer-driven harm reduction services for PWSDs in the rural Abbotsford and urban Vancouver communities. We hypothesized that the differences in the concentrations of harm reduction programs in the two cities would be reflected in the level of harm promoting and reducing behaviours of PWSDs. In addition, number of people within networks of PWUIDs have been shown to play a significant role in influencing risky drug behaviours [17–19]. We analysed network size in order to determine whether those in larger networks shared equipment with more people than those who had smaller egocentric networks, in both communities. Through the mapping of social networks, we determined whether there was an association between the availability of peer-led harm reduction programs and the size of the networks of PWSD. Results of this study would allow us to describe the individuals residing in these communities; their harm reduction needs, and potential gaps in existing community programs.

## Methods

This study was done in collaboration with the University of British Columbia and the drug using communities of Abbotsford and the DTES in Vancouver, British Columbia. Researchers used a community-based participatory action research (CBPAR) approach that engages the community in the development, implementation, and dissemination of research.

Involvement of board members of VANDU was continuous throughout the project. (EJ) had worked on a previous social network project and one of the VANDU board members suggested he build on that work to include people who smoke drugs, as many of them visited VANDU to obtain clean pipes, but not a lot was known about them. EJ’s postdoctoral fellowship application was successful and VANDU board members requested to play a greater role in data collection as peer interviewers, and be paid for their time. They reviewed drafts of the study design, questionnaire and consent from May through August of 2016, and provided helpful advice on recruitment and retention of peer interviewers. Suggestions included paying peer interviewers for each questionnaire completed, conducting quality checks, and asking questions about opioid use, as the overdose epidemic was just entering its early phase. Last, they suggested it was important to study rural drug use and introduced EJ to the BC-Yukon Association of Drug War Survivors (BC-YADWS) where he met co-author ET, a peer supervisor in Abbotsford. BC-YADWS also reviewed study implementation; training and data collection plans. Co-authors ET and SM recommended and supervised peers who were able to conduct interviews. EJ has continually consulted with the peer researchers at each draft, who recommended submitting to Journal of Harm Reduction, as the contents are freely available to members of the public.

After consultations with two community groups above, the research proposal was approved, along with the questionnaire, and oral consent. Ethics approval for the research protocol and consent form was obtained from the University of British Columbia (H16-01580) and from the University of Ottawa (H-05-18-741). Posters were distributed by community members in Vancouver and study staff handed out advertisements for peer researchers in Abbotsford, both of which emphasised hiring community members. Eight and seven people who smoke drugs with lived drug experiences had responded to the advertisements disseminated in both Vancouver and Abbotsford, respectively. They were interviewed, evaluated and recommended by SM and ET, above, and were prepared to undergo ethics and interviewer training. They were recruited and paid $20.00 per hour, similar to research assistants at the university. We used a respondent-driven sampling (RDS) approach to better reach people who smoke street drugs and are usually marginalised by health care workers and researchers. This method assumes that a representative sample of the population is obtained following approximately six waves of chain-referrals, after which equilibrium of proportions of characteristics is achieved [20]. These peer researchers were asked to recruit 10 “contacts or friends” in their networks, who used illegal drugs mainly through smoking; were 19 years of age or older, and to whom they felt comfortable administering the questionnaire [21]. Besides providing information on themselves, each of the 10 friends then provided proxy information on ten of their “friends or contacts” and the relationships between them, for a total of about 1500 people. However, recruitment ended at the first wave of contacts or friends, as this was a pilot to demonstrate feasibility of using CBPAR and RDS rather than to provide generalizable results. Each peer researcher completed 10 h of training in applied ethics.

The pilot was essential as CBPAR is challenging in it itself, and in this study, participants not only guided the direction of the research, but also underwent ethics and informed consent training, crafted and administered the questionnaire, entered data and commented on the draft papers. Additionally, network questionnaires can be very time consuming as the numbers of network members nominated multiplies the number of questions asked about each one, and respondents curtail their responses accordingly. In most studies in Canada, respondents are asked to nominate three friends who may then contact study personnel and if they consent, are recruited. Here, each peer researcher interviewed 10 friends (alters) and asked them questions about 10 of their friends, resulting in many questions and long interviews.

The questionnaire which each peer administered to each of their 10 friends was divided into two parts. The first included information from each friend, on housing and place of residence; age, gender, ethnic origins, drug smoking behaviours, including sharing equipment and frequency and type of drug smoked, medical conditions, overdosing; experience of violence, frequency of health care provider visits, mental health, injuries to the mouth as a result of smoking drugs, and drug smoking equipment. The second part of the questionnaire asked each of the 10 friends to list 10 of their friends whom they knew smoked drugs. For each one, questions were asked on; demographic and housing information, length of time that they have known the person, medical conditions, how close they were to the person, and smoking and injecting behaviours. Next, they were asked to select a from a list of roles or actions which one they considered the most valuable. For example, their friend had “taught me to fix my pipe or dope”, or had “administered naloxone when I overdosed”. Last, they were asked to fill in a grid of which friends knew each other, and how close they were; close, somewhat close, or not very close.

Because many participants used aliases in lieu of legal names, egocentric network sizes were determined using a hierarchical cross-network matching algorithm. The first set of 10 friends of a peer researcher was added to the lists of the 10 which each their friends reported, resulting in a longer list. Names, aliases and demographic variables which matched were considered to refer to the same person. A second level of comparisons were based on; age (within ten-year range), drug(s) of choice, current drug use status, and years of drug use (within a five-year range) [16]. Records matching on three or more were considered to belong to the same individual. Physical and mental health status, and routes of drug administration were used to verify matches and to resolve discrepancies.

All variables with missing data exceeding 10% were divided into two categories. We defined missing data as incomplete, unclear, or ‘don’t know’ responses, and added in the unknown responses due to the extremely low number of participants selecting this option. The association between missing data and the remaining variables was tested using Chi-squares or Fisher’s exact tests. Bivariate analyses comparing participants in Abbotsford and the DTES were conducted using the same two tests with pairwise deletion for missing values.

Statistical analysis was conducted using SAS Software Version 9.4 and network analyses were conducted on UCINET 5.1. Networks were visualized using Organizational Risk Analyzer.

## Results

**Fig. 1:**
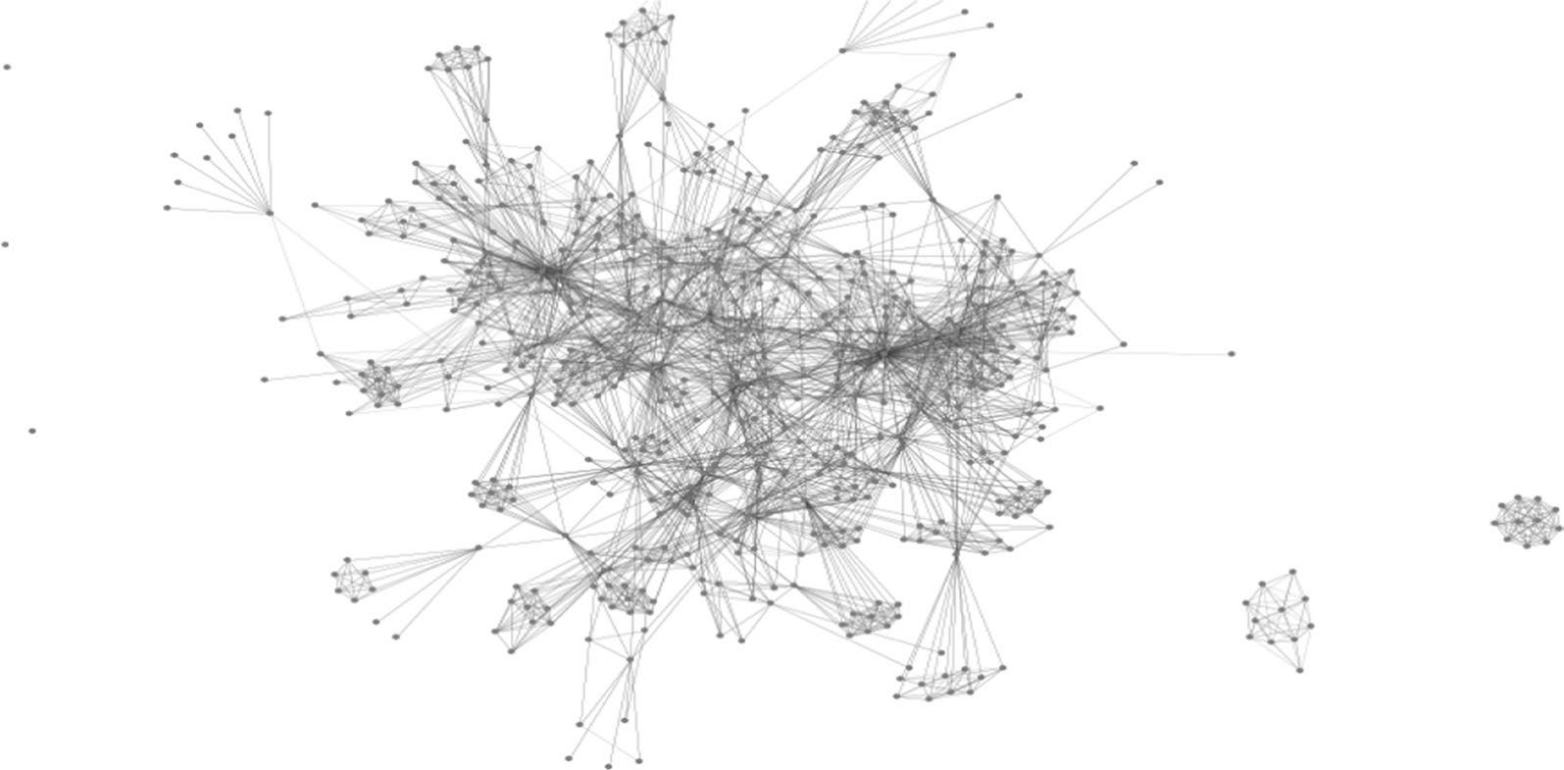
Social network of eight abbotsford participants, 79 recruits, and their 739 friends. Dots represent individuals and lines between them relationships, including recruitment referrals into the study

**Fig. 2:**
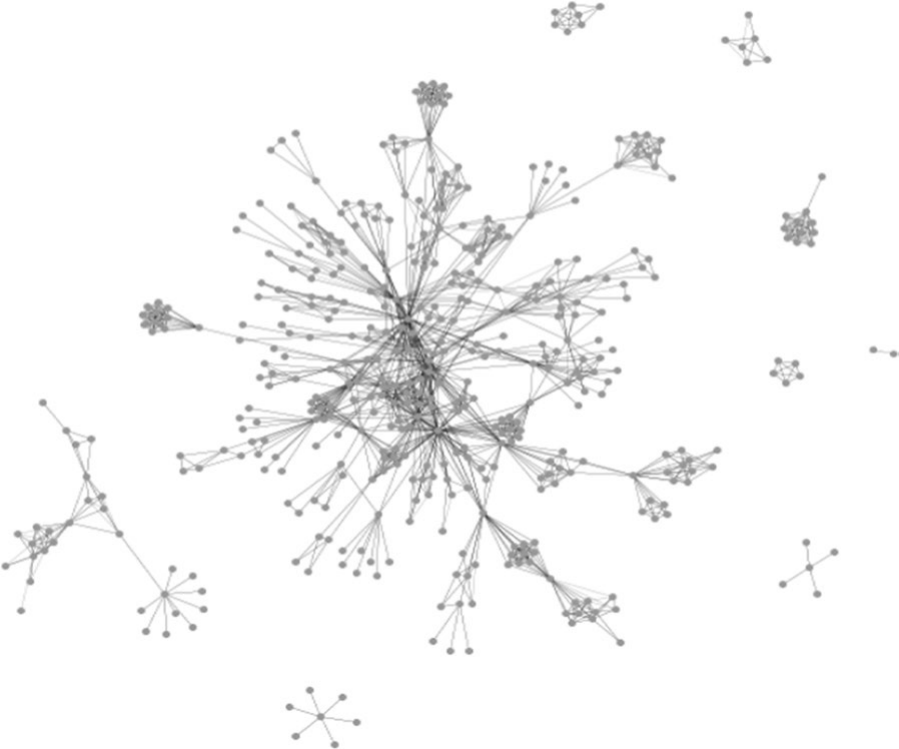
Social network of seven DTES participants, 70 recruits, and their 498 friends. Dots represent individuals and lines between them relationships, including recruitment referrals into the study

Eight and seven peer researchers in Abbotsford and Vancouver recruited 79 and 70 friends and contacts, (alters) who reported on 739 and 498 friends, respectively (Figs. 1, 2).

The training and retainment of peer researchers has already been described; 8 of 10 completed 10 h of ethics training from Abbottsford, and 6 of 7 from DTES [21]. Although one participant in Abbotsford did not complete the questionnaire, all other peer researchers recruited 10 participants each for a total of 149 alters. Because there was substantial demographic and behavioural data missing, we compared the records with responses to those without, in order to clarify possible biases (Table 1).

**Table 1:** Demographic and behavioural correlates of missing responses greater than 10% for Vancouver and Abbotsford from 149 participants

Variable with missing values above 10%	Demographic or behavioural characteristic	Missing values *N* (% or mean)	Non-missing values *N* (%)	*P *value
Number of people	Homeless	11(61)	40(30)	0.01
	Arrested for smoking or using illicit drugs in the public	6(33)	18(13)	0.04
Average number illicit drugs smoked/used per day	experienced psychosis or paranoia as a result of smoking illicit drugs	11(100)	80(58)	0.01
Number of times lent, borrowed, or shared pipes	Have lent, borrowed, or shared pipes	11(100)	73(53)	< 0.01
	Used meth equipment for pipe screens	7(64)	41(30)	0.02
Number of experiences of violence or exploitation by other people who use drugs	Have experienced violence or exploitation by other people who use drugs	15(94)	68(52)	< 0.01
	Have experienced violence or exploitation when using drugs in public by the police	11(69)	37(28)	< 0.01
	Have experienced violence or exploitation when using drugs in public by drug dealers	12(75)	45(34)	< 0.01
Number of experiences of violence or exploitation when using drugs in public by the police	Arrested for smoking or using illicit drugs in the public	6(50)	18(13)	< 0.01
	Received tickets for smoking or using illicit drugs	5(38)	7(5)	< 0.01
	Have experienced violence or exploitation by other people who use drugs	11(92)	72(53)	< 0.01
	Have experienced violence or exploitation when using drugs in public by the police	9(75)	39(29)	< 0.01
	Have experienced violence or exploitation when using drugs in public by drug dealers	11(92)	46(34)	< 0.01
Number of experiences of violence or exploitation when using drugs in public by drug dealer	Age at survey	12(38)	130(45)	0.04
	Arrested for smoking or using illicit drugs in the public	5(42)	19(14)	0.03
	Have experienced violence or exploitation by other people who use drugs	11(92)	72(53)	0.01
	Have experienced violence or exploitation when using drugs in public by the police	11(92)	37(27)	< 0.01
	Have experienced violence or exploitation when using drugs in public by drug dealers	12(100)	45(33)	< 0.01
Number of experiences of psychosis or paranoia as a result of smoking illicit drugs in the past	Have experienced psychosis or paranoia as a result of smoking illicit drugs	20(100)	72(55)	< 0.01
Number of days since visiting a doctor or nurse	Have experienced psychosis or paranoia as a result of smoking illicit drugs	1(11)	91(65)	< 0.01
	Have experienced violence or exploitation by other people who use drugs	2(22)	81(58)	0.04
	Have experienced violence or exploitation when using drugs in public by drug dealers	0(0)	57(41)	0.01
Average amount of money spent on drugs	Have lent, borrowed, or shared pipes	2(18)	82(59)	0.01

As above in our study aims, we compared to Abbotsford and DTES participants. A greater proportion of participants from DTES were male (61% vs. 37%); self-identified as First Nations (aboriginal Canadian), (58% vs. 28%); and reported living in supported housing (50% vs. 13%). In Abbotsford, more participants reported living with friends and family (16% vs. 3%), (Table 2). There was no statistically significant difference in the proportion of self-reported medical conditions between participants in Abbotsford and DTES.

**Table 2:** Participant characteristics by location *n* = 149, presented as either number of participants (percent frequency) or mean (standard deviation)

Demographic variables of participants	Abbotsford (n = 79)	DTES (n = 70)	*P* value
	*n* or mean (% or SD)	*n* or mean (% or SD)	
Age	43(10)	46(12)	0.12
Male	29(37)	42(61)	< 0.01*
Homeless	32(41)	19(27)	0.09
House/apartment	24(30)	14(20)	0.15
Living with friends or family	13(16)	2(3)	0.01*
Supported living	10(13)	35(50)	< 0.01*
First Nations	22(28)	40(58)	< 0.01*
Relationship status	16(20)	20(29)	0.24
*Medical condition*			
HCV	36(46)	23(33)	0.11
No medical condition	29(37)	32(46)	0.26
Anxiety	42(53)	37(53)	0.97
Depression	49(62)	35(50)	0.14
No mental conditions	12(15)	13(19)	0.58
Other mental conditions	41(52)	31(44)	0.35
*Drug use*			
Meth	61(77)	38(54)	< 0.01*
Crack	29(37)	42(60)	< 0.01*
*Pipe source*			
Outreach organizations	60(76)	62(89)	0.05*
Store	23(29)	7(10)	< 0.01*
Peers	19(24)	2(3)	< 0.01*
Lend, borrow, or shared pipes	59(75)	25(36)	< 0.01*
Overdosed in the past month	11(14)	4(6)	0.11
Trained on how to use naloxone (Narcan)	43(54)	57(81)	< 0.01*
Trained on CPR	46(58)	56(80)	< 0.01*
Carry naloxone	37(47)	33(47)	0.97
Have rescued peers who have overdosed	32(41)	34(49)	0.28
Arrested for smoking or using illicit drugs in public	14(18)	10(14)	0.57
Received tickets for smoking or using illicit drugs	5(6)	7(10)	0.41
*Have experienced violence or exploitation when using drugs in public by*			
Users	48(61)	35(50)	0.19
Dealers	31(39)	26(37)	0.79
Police	27(34)	21(30)	0.59
Have experienced psychosis or paranoia as a result of smoking illicit drugs	51(65)	40(57)	0.35
Have had blisters, cuts, damage, or infections to mouth, oral area or lips in the last month	27(34)	12(17)	0.02*
Number of days since visiting a doctor or a nurse	249(585)	47(91)	0.01*
Public drug use	69(87)	37(53)	< 0.01*
*Pipe screen material*			
Brillo	27(34)	32(46)	0.15
Brass	28(35)	26(37)	0.83
Meth equipment	31(39)	17(24)	
*Network characteristics*			
Network size	23(14)	19(14)	0.11

*Statistical difference *p* < 0.05

Participants from Abbotsford preferred smoking methamphetamine (77% vs. 54%), but DTES participants preferred crack cocaine (60% vs. 37%). Abbotsford participants were more likely to acquire pipe paraphernalia from stores (29% vs. 10%) and from peers (24% vs. 3%) than participants in DTES, who were more likely to acquire pipe paraphernalia from outreach organizations (89% vs. 76%). Additionally, a greater proportion of Abbotsford participants reported lending, borrowing, or sharing pipes (75% vs. 36%); having had blisters, cuts, damage, or infections in the mouth, oral area, or lips (34% vs. 17%); using meth equipment for pipe screens (39% vs. 24%); and engaging in public drug use (87% vs. 53%), compared with those from DTES. More DTES participants reported being trained on how to use naloxone (81% vs. 54%) and CPR (80% vs. 58%). Finally, the mean number of days since visiting a doctor or nurse was significantly lower for participants in DTES than for participants in Abbotsford.

Compared with contacts of DTES participants, a greater proportion of contacts of Abbotsford participants were reported as homeless (48% vs. 17%), HCV positive (20% vs. 13%), mentally ill (27% vs. 11%), tobacco smokers (94% vs. 90%), and methamphetamine users (81% vs. 50%) (Table 3). In contrast, contacts of DTES participants were more likely to report being male (61% vs. 53%), living in supported housing (34% vs. 16%), and preferring crack cocaine (43% vs. 25%) than contacts of Abbotsford participants.

**Table 3:** Distribution of contact characteristics according to location

Demographic variables of contacts	Abbotsford (*n* = 739)	DTES (*n* = 498)	*P *value
	*n* (%)	*n* (%)	
Male	393(53)	304(61)	< 0.01*
Age	41.99(34)	43(10)	0.69
Homeless	357(48)	87(17)	< 0.01*
Years living in Abbotsford	23(3)	4(1)	0.01*
House/apartment	201(27)	31(6)	< 0.01*
Living with friends or family	14(2)	3(1)	0.08
Years living in DTES	0(0)	177(36)	< 0.01*
Supported living	115(16)	168(34)	< 0.01*
*Medical conditions*			
Mental Illness	196(27)	55(11)	< 0.01*
HIV	41(6)	22(4)	0.38
HCV	149(20)	63(13)	< 0.01*
*Drug use*			
Current drug user	714(97)	468(96)	0.38
Injection drug use	386(52)	280(56)	0.17
Smoking drug use	694(94)	449(90)	0.01*
Meth	596(81)	249(50)	< 0.01*
Crack	183(25)	213(43)	< 0.01*
Opioids	346(47)	222(45)	0.44

*N* = 1386 contacts. Data presented as either percent frequency or mean (standard deviation)

*Statistical difference *p* < 0.05

## Discussion

The training and retention of peer researchers and the success of community peer involvement is reflected in the number of participants recruited and the completion of questions about each of 10 additional alters. Table 1 shows the clusters of questions which people tended not to answer, in which people were asked repeatedly to remember numbers of events, which is difficult to remember and tiring. For example, answers to three consecutive questions about frequency of violence experienced by PWSD, from police, dealers and other PWSD decreased substantially from the first to the third question. Simple corrections such as interspersing questions containing numbers of events with other questions an omitting some can be made in future research. To our knowledge this is the first study combining CBPAR and RDS in people who use drugs, and we believe that the high response rate of 149 participants is a reflection of the value placed on personal relationships within the community of PWSD. The high number of participants referred by initial peer researchers has demonstrated beyond a doubt that the usual three recruits will be feasible [22] resulting in the optimal several waves of recruiters and respondents [17]

The disparity in allocated resources is reflected in the socio-demographic and behavioural attributes of the participants and their listed contacts. Participants in Abbotsford were more likely to report engaging in harmful drug behaviours such as sharing, lending, or borrowing pipes and smoking in public areas, and less likely to report harm reducing activities, such as training on use of naloxone and CPR, carrying naloxone, and acquiring pipes from outreach organizations.

Fifty percent of DTES participants reported residing in a supported living environment, including single room occupancy (SRO) hotels and aboriginal housing. Participants in Abbotsford, however, reported a statistically higher percentage living with friends or families. Likewise, contacts of Abbotsford participants were more likely to be homeless or living in private housing while contacts of DTES participants were more likely to live in a supported living environment. This is consistent with previous research on the housing trends of drug users in DTES, where single occupancy housing was highly accessible. A 2015 survey of SRO hotels in DTES revealed a vacancy rate of only 4% among the 4379 and 9645 private and non-profit SRO units in the community [23]. Similarly, Shannon et al. reported that 70% of recruited DTES residents reported residing in SRO hotels and aboriginal housing [24].

The comparatively fewer harm reduction services in Abbotsford is likely a factor in the greater prevalence of participants’ contacts with HCV and mental illness. There was a significantly greater percentage of contacts (7.51%) in Abbotsford who reported being HCV positive compared to DTES contacts. The higher rates of infection are consistent with higher rates of reported pipe sharing and oral blisters, cuts, or sores among participants in Abbotsford. All of these are known to facilitate the transmission of bloodborne infections. The reasons for the difference in HCV infection rate, which was significant only among contacts and not participants, may be due to the small sample size, under-reporting of events due to social desirability bias, or unknown serostatus.

Over 77% of participants and 80% of contacts in Abbotsford reported consuming methamphetamine, whereas DTES participants and their contacts indicated crack as their drug of choice. Abbotsford participants were more likely to be female and Caucasian, consistent with previous studies where, relative to cocaine users, users of methamphetamine are more likely to be female and Caucasian [25, 26]. The greater use of methamphetamine may be a consequence of Abbotsford’s proximity to the United States border. Additionally, a greater number of participants in Abbotsford were female because recruitment posters were distributed in Warm Zone-Women’s Resource Society, whereas VANDU caters to both women and men who use drugs.

Although participants in Abbotsford had larger mean network sizes, this was statistically insignificant. This suggests that urbanization has no influence on the network size of people who smoke illicit drugs. Previous research on network structure has identified multiple individual-level factors that are associated with network size, including age, gender, and education level [27]. Research into the network composition of urban versus rural social networks found urban residents to generally be socially isolated and rural residents to be socially connected and highly involved [28, 29]. However, Hooghe and Botterman found that among residents in Belgium, the population density and size of a region had no relationship to the quantity and degree of social association between residents [30]. Furthermore, users of illicit drugs are more likely to belong to a low socioeconomic class, and poverty has been associated with an increased sense of cohesion that may negate the influence of urbanization [31]. In addition, it is likely that because drug use is considered undesirable in North American society, all PWSD become part of networks of similar size and density, and marginalised from main stream society.

This study consisted of a single wave of chain-referrals through initial key informants, to demonstrate feasibility of the community-based participatory approach in conjunction with respondent driven sampling. Subsequent waves of referred participants would be preferable for the study population to be considered representative of the DTES and Abbotsford drug smoking community.

Nevertheless, the accuracy of participants’ recollection of their contacts’ behavioural and demographic characteristics can be assumed to be fairly accurate. Romney and Weller demonstrated that individuals who frequently interact with each other are a reliable source of information [32]. Barrera and Arnold reported a high correlation (*r* = 0.88) between test and retest reporting of social network members [33, 34]. Hammer observed a recall rate of 79% for contacts seen more than once a week [35]. Participants in Sudman’s 39-person study were able to recall 92% of close contacts [36], and Brewer found that injecting drug users remembered 78% of their drug using partners [37]. However, because responses were self-reported, they may have been subject to the social desirability bias.

## Conclusion

Respondent driven sampling used in conjunction with CB-PAR was successful in this context, where community-based organisations were entrenched and supportive. The fact that EJ, (co-author) had a long and trusting relationship with community groups, added to its success. While these two factors played a large part in the projects’ success, projects like this one are challenging and depend also on the local harm reduction environment and interactions, making generalisations near impossible. There are key differences in the demographic and behavioural traits between PWSD in Abbotsford and DTES, which indicate the greater emphasis on harm reduction in DTES relative to Abbotsford. Recruited participants in DTES were found to engage in more harm reducing behaviours than Abbotsford participants, where harm reduction initiatives are limited. We recommend that closer attention be paid to Abbotsford and other rural regions across Canada that have traditionally suffered from lack of effective harm reduction programs.

## Data Availability

The data may be available under request to Dr. Ehsan Jozaghi.
